# Influence of Amino Acid Compositions and Peptide Profiles on Antioxidant Capacities of Two Protein Hydrolysates from Skipjack Tuna (*Katsuwonus pelamis*) Dark Muscle

**DOI:** 10.3390/md13052580

**Published:** 2015-04-27

**Authors:** Chang-Feng Chi, Fa-Yuan Hu, Bin Wang, Zhong-Rui Li, Hong-Yu Luo

**Affiliations:** 1 National Engineering Research Center of Marine Facilities Aquaculture, School of Marine Science and Technology, Zhejiang Ocean University, 1st Haidanan Road, Changzhi Island, Lincheng, Zhoushan 316022, China; E-Mail: moonriveryue@163.com; 2 Zhejiang Provincial Engineering Technology Research Center of Marine Biomedical Products, School of Food and Pharmacy, Zhejiang Ocean University, 1st Haidanan Road, Changzhi Island, Lincheng, Zhoushan 316022, China; E-Mail: lisa8919@163.com; 3 Division of Life Science, Hong Kong University of Science and Technology, Clear Water Bay, Kowloon, Hong Kong, China; E-Mail: zlibb@ust.hk

**Keywords:** skipjack tuna (*Katsuwonus pelamis*), dark muscle byproduct, protein hydrolysate, peptide, antioxidant activity

## Abstract

Influence of amino acid compositions and peptide profiles on antioxidant capacities of two protein hydrolysates from skipjack tuna (*Katsuwonus pelamis*) dark muscle was investigated. Dark muscles from skipjack tuna were hydrolyzed using five separate proteases, including pepsin, trypsin, Neutrase, papain and Alcalase. Two hydrolysates, ATH and NTH, prepared using Alcalase and Neutrase, respectively, showed the strongest antioxidant capacities and were further fractionated using ultrafiltration and gel filtration chromatography. Two fractions, Fr.A3 and Fr.B2, isolated from ATH and NTH, respectively, showed strong radical scavenging activities toward 2,2-diphenyl-1-picrylhydrazyl radicals (EC_50_ 1.08% ± 0.08% and 0.98% ± 0.07%), hydroxyl radicals (EC_50_ 0.22% ± 0.03% and 0.48% ± 0.05%), and superoxide anion radicals (EC_50_ 1.31% ± 0.11% and 1.56% ± 1.03%) and effectively inhibited lipid peroxidation. Eighteen peptides from Fr.A3 and 13 peptides from Fr.B2 were isolated by reversed-phase high performance liquid chromatography, and their amino acid sequences were determined. The elevated antioxidant activity of Fr.A3 might be due to its high content of hydrophobic and aromatic amino acid residues (181.1 and 469.9 residues/1000 residues, respectively), small molecular sizes (3–6 peptides), low molecular weights (524.78 kDa), and amino acid sequences (antioxidant score 6.11). This study confirmed that a smaller molecular size, the presence of hydrophobic and aromatic amino acid residues, and the amino acid sequences were the key factors that determined the antioxidant activities of the proteins, hydrolysates and peptides. The results also demonstrated that the derived hydrolysates and fractions from skipjack tuna (*K. pelamis*) dark muscles could prevent oxidative reactions and might be useful for food preservation and medicinal purposes.

## 1. Introduction

Oxidative deterioration is a prominent threat to the quality of lipid-containing foods that produces off-flavors and reduces the nutritive value and safety of the foods by forming secondary reaction products during processing and storage [1,2,3]. In addition, the generation of radicals, especially reactive oxygen species (ROS), during oxidative stresses can react with substances in the body, resulting in cellular damage and disorders [4,5]. Antioxidants can inhibit oxidative reactions by scavenging free radicals and interrupting the radical chain reaction of lipid peroxidation. Therefore, the elimination of ROS and other free radicals is considered one of the most important antioxidant mechanisms in food and biological systems. Synthetic antioxidants, such as butylated hydroxytoluene (BHT) and butylated hydroxyanisole (BHA), are used to overcome the deleterious effects induced by free radicals. However, their use has been strictly regulated due to potential health hazards [6,7]. Fortunately, antioxidant peptides obtained from food proteins can serve as potential substitutes for synthetic antioxidants because of their elevated activities and stabilities [8,9].

Traditionally, dark muscle byproducts from the seafood industry are primarily used to produce low-market-value products, such as fishmeal and fertilizer, because of their dark color, oxidation, off-flavors and poor functional properties [10]. There are multiple options for recovering dark muscle byproducts and converting them to value-added products [11,12]. Among these possibilities, enzymatic hydrolysis is one of the most efficient methods for the high-value use of dark muscle byproducts [13,14], and some antioxidant peptides have been isolated and identified from the muscles of various aquatic species. Jiang *et al.* purified two antioxidant peptides (HDHPVC and HQKVC) from round scad muscle protein hydrolysate, and their studies indicated that the two novel peptides could be developed into antioxidant ingredients in functional foods [11]. Peptides of QWPAQ, FLHRP, and LMGQW, isolated from the protein hydrolysates of monkfish muscle [15], and LDK, WDR and PYFNK, isolated from the hydrolysates of *Sphyrna lewini* muscle protein [3,16], were effective against lipid peroxidation and exhibited good scavenging activity on 2,2-diphenyl-1-picrylhydrazyl (DPPH) radicals (DPPH•), hydroxyl radicals (HO•), and superoxide anion radicals (${\text{O}}_{\text{2}}^{-}$•) in dose-dependent manners. VCSV and CAAP from the protein hydrolysates of flounder fish muscle showed good scavenging activity against DPPH• and high cytoprotective activities against 2,2-azobis-(2-amidinopropane) dihydrochloride without significant cytotoxicity and scavenged total reactive oxygen species in Vero cells [17]. Nonapeptide LGLNGDDVN, purified from the tryptic hydrolysates of conger eel muscle protein, effectively scavenged HO• and carbon-centered radicals and performed better than the natural antioxidant, α-tocopherol, for the prevention of lipid peroxidation *in vitro* [18]. DLDLRKDLYAN, from the protein hydrolysates of defatted skipjack roe, showed strong metal chelating activities as well as 2,2′-azino-bis(3-ethylbenzthiazoline)-6-sulfonic acid (ABTS) radical and singlet oxygen scavenging activities and could be further employed as a functional food ingredient [19]. The peptide LSGYGP, isolated from tilapia gelatin, could alleviate the UV-induced abnormal changes in antioxidant indicators, protect skin lipids and collagen, and repair the synthesis of endogenous collagen in a dose-dependent manner; thus, it could be a novel anti-photoaging agent from natural resources [20]. Hexapeptide WCTSVS, from the hydrolysates of the edible parts of the Indian squid, exhibited strong free radical scavenging, metal chelation and reducing power. It also exhibited no cytotoxic effects on breast cancer cells (MCF7) and scavenged reactive oxygen species at the cellular level under H_2_O_2_-induced stress [21,22]. These studies indicate that aquatic muscle proteins are high-quality raw materials that can be used for the preparation of antioxidant peptides. The peptides derived from the protein hydrolysates of seafood muscles could serve as potent antioxidants against oxidative stresses and be used as an efficient and safe additive in food processing.

China is among the world’s largest producers and exporters of canned tuna. Skipjack tuna (*K. pelamis*) is the most viable commercial species in the tuna industry. In 2013, more than 100 tons of raw tuna were processed in Zhoushan City, Zhejiang Province of China. Approximately 50% of the processed raw materials were considered industrial byproducts, such as dark muscle, head, skin, bones and viscera [23]. Recently, several studies have reported on the use of tuna byproducts through enzymatic hydrolysis [12,23,24,25,26,27,28]. Several bioactive peptides, including GDLGKTTTVSNWSPPKYKDTP, PVSHDHAPEY, PSDHDHE, VHDY, VKAGFAWTANQQLS, LPTSEAAKY and PMDYMVT, have been extracted from the protein hydrolysates of tuna frames [24], cooking juices [25], backbones [26], and dark muscles [27]. These studies provide useful information for the preparation of peptides from tuna byproducts and for converting these byproducts into value-added peptide products with significant functional and nutritional properties by enzymatic hydrolysis. However, more research must be performed to further utilize the dark muscles of tuna. Therefore, in the present study, skipjack tuna dark muscle (STDM) samples were hydrolyzed using five separate proteases. The two protein hydrolysates with the highest antioxidant activities were fractionated using ultrafiltration and gel filtration chromatography. The influence of amino acid compositions and peptide profiles on antioxidant activities of the resulting fractions was comparatively analyzed.

## 2. Results and Discussion

### 2.1. Preparation of Protein Hydrolysates of STDM Using Five Proteases

Radical scavenging assays are fast, convenient, and efficient in predicting the antioxidant activities of protein hydrolysates, their fractions, and purified peptides. Therefore, the relatively stable radicals DPPH•, HO•, and ${\text{O}}_{\text{2}}^{-}$• have been widely used to test the ability and evaluate the antioxidant activity of compounds to act as free radical scavengers or hydrogen donors [7]. In addition, the degree of hydrolysis (DH) could affect the molecular sizes and amino acid compositions of the peptides and thereby affect the biological activities of the peptides formed during hydrolysis. Therefore, the DH is an important parameter in determining the functional properties of protein hydrolysate preparations [29]. In this experiment, radical scavenging assays and the DH were used to determine the hydrolytic capacities of proteases on defatted STDM.

**Figure 1:**
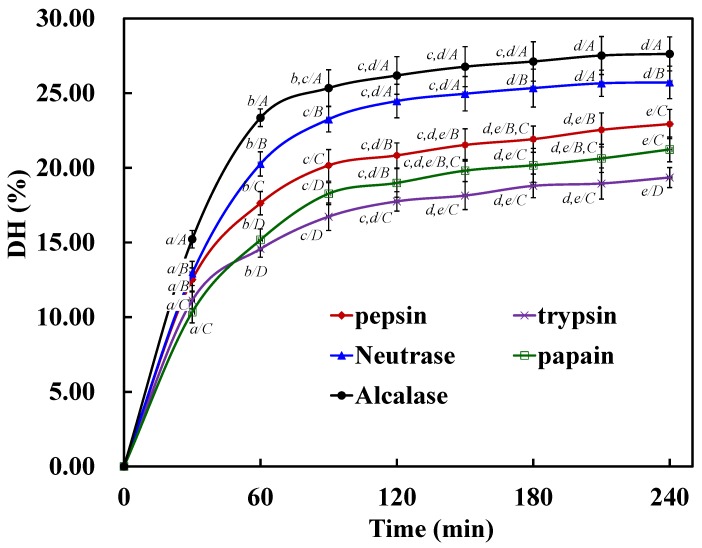
DH of protein hydrolysates of STDM using five proteases. All values mean ± SD. (*a*–*e*) Values with different letters indicated significant differences in the same sample at different times (*p <* 0.05); (*A*–*D*) Values with different letters indicated significant differences in different samples at the same time (*p* < 0.05).

Defatted STDM samples were separately hydrolyzed with pepsin, trypsin, Neutrase, papain and Alcalase. The DH values and radical (DPPH•, HO•, and ${\text{O}}_{\text{2}}^{-}$•) scavenging activities of the resulting hydrolysates were shown in Table 1 and Figure 1. After 4 h of hydrolysis, the Alcalase hydrolysate (ATH) showed a significantly higher DH value (*p* < 0.05) of 27.63% ± 1.14% followed by the Neutrase hydrolysate (NTH) (25.72% ± 1.09%), whereas the trypsin hydrolysate showed a significantly lower DH value (*p* < 0.05) of 19.35% ± 0.67%. The DH curves were similar to those previously reported for the hydrolysates from zebra blenny proteins [30] and sole, squid [31], and cuttlefish skin gelatins [32].

The DPPH•, HO•, and ${\text{O}}_{\text{2}}^{-}$• scavenging activities of the five hydrolysates are shown in Table 1. ATH exhibited significantly higher DPPH•, HO•, and ${\text{O}}_{\text{2}}^{-}$• scavenging activities (*p* < 0.05), with EC_50_ values of 4.54 ± 0.43, 1.27 ± 0.12, and 5.67 ± 0.26 mg/mL, respectively. NTH followed with EC_50_ values of 5.38 ± 0.15, 1.58 ± 0.11, and 6.38 ± 0.53 mg/mL, whereas the lowest radical scavenging activities were observed in the trypsin hydrolysates. This data trend was in agreement with the DH order of the five hydrolysates. Based on these results, ATH and NTH were selected for follow-up studies.

**Table 1:** Radical (DPPH•, HO•, and ${\text{O}}_{\text{2}}^{-}$•) scavenging activities and the degree of hydrolysis (DH, %) of the protein hydrolysates of STDM using five proteases.

Enzyme	DH (%)	EC_50_ (mg/mL) of Protein Hydrolysates		
		DPPH•	HO•	${\text{O}}_{\text{2}}^{-}$
Pepsin	22.93 ± 0.98 ^a^	7.09 ± 0.37 ^a^	2.37 ± 0.09 ^a^	7.65 ± 0.42 ^a^
Trypsin	19.35 ± 0.67 ^b^	9.36 ± 0.51 ^b^	4.09 ± 0.16 ^b^	9.37 ± 0.40 ^b^
Neutrase	25.72 ± 1.09 ^c^	5.38 ± 0.15 ^c^	1.58 ± 0.11 ^c^	6.38 ± 0.53 ^c^
Papain	21.23 ± 0.83 ^a^	8.65 ± 0.57 ^b^	3.42 ± 0.23 ^d^	8.66 ± 0.48 ^b^
Alcalase	27.63 ± 1.14 ^d^	4.54 ± 0.43 ^d^	1.27 ± 0.12 ^e^	5.67 ± 0.26 ^c^

All values were the mean ± standard deviation (SD). EC50 (mg/mL) was defined as the concentration at which a sample caused a 50% decrease in the initial concentrations of DPPH•, HO•, and ${\text{O}}_{\text{2}}^{-}$•. (a–e) Values with different letters indicated significant differences in the same sample at different times (*p* < 0.05).

### 2.2. Isolation of Antioxidant Peptides

#### 2.2.1. Ultrafiltration (UF) Fractionation of ATH and NTH

UF is a membrane filtration process in which forces pressure or concentration gradients lead to a separation through a semipermeable membrane. Currently, UF membrane technology has been of great importance for the purification, concentration, and fractionation of several products in various areas, such as the food, pharmaceutical, and biotechnology industries, and has been one of the best available techniques for the enrichment of peptides [33]. ATH-I (MW ˃3 kDa) and ATH-II (MW ˂3 kDa) fractionated from ATH, and NTH-I (MW >3 kDa) and NTH-II (MW <3 kDa) from NTH were prepared using a 3 kDa molecular weight cut-off (MWCO) membrane. ATH, NTH and their four fractions scavenged DPPH•, HO•, and ${\text{O}}_{\text{2}}^{-}$• in a concentration-dependent manner (data not shown), and their EC_50_ values are shown in Table 2.

The results indicated that ATH-II exhibited significantly higher antioxidant activity than ATH and ATH-I (*p* <0.05), with EC50 values of 2.21 ± 0.12, 0.58 ± 0.05, and 2.74 ± 0.11 mg/mL for DPPH•, HO•, and ${\text{O}}_{\text{2}}^{-}$•, respectively. Similarly, NTH-II showed significantly higher antioxidant activity than NTH and NTH-I (*p* <0.05), with EC50 values of 3.09 ± 0.23, 0.85 ± 0.06, and 2.83 ± 1.08 mg/mL for DPPH•, HO•, and ${\text{O}}_{\text{2}}^{-}$•, respectively. The results suggested that the type of peptide played a key role in the antioxidant activity of the protein hydrolysates and their fractions. The original hydrolysate was filtered with a 3 kDa MWCO membrane, which removed high MW peptides. Therefore, the resultant hydrolysate fractions with high bioactivities were composed of low MW peptides [5,34]. These results agreed with the previous report that protein hydrolysates or peptides with lower MWs were observed to have higher antioxidant activities and more effectively interact with radicals, thus intervening in the oxidation process [3,35].

**Table 2:** Radical (DPPH•, HO•, and ${\text{O}}_{\text{2}}^{-}$•) scavenging activities of protein hydrolysates (ATH and NTH) and their fractions prepared by ultrafiltration and gel filtration chromatography.

Sample	EC_50_ (mg/mL)		
	DPPH•	HO•	${\text{O}}_{\text{2}}^{-}$
**ATH and Its Fractions**			
**ATH**	4.54 ± 0.43 ^a^	1.27 ± 0.12 ^a^	5.67 ± 0.26 ^a^
**ATH-I**	7.06 ± 0.63 ^b^	1.56 ± 0.17 ^b^	6.82 ± 0.47 ^b^
**ATH-II**	2.21 ± 0.12 ^c,f^	0.58 ± 0.05 ^c,d^	2.74 ± 0.11 ^c^
**Fr.A1**	3.27 ± 0.24 ^d,e^	0.73 ± 0.08 ^d,e^	3.54 ± 0.26 ^d^
**Fr.A2**	2.42 ± 0.15 ^f^	0.55 ± 0.06^c,d,f^	2.76 ± 0.18 ^c^
**Fr.A3**	1.08 ± 0.08 ^g^	0.22 ± 0.05 ^g^	1.31 ± 0.11 ^e^
**Fr.A4**	1.76 ± 0.04 ^c^	0.37 ± 0.03 ^f,g^	1.69 ± 0.09 ^e^
**NTH and Its Fractions**			
**NTH**	5.38 ± 0.15 ^h^	1.58 ± 0.11 ^b^	6.38 ± 0.53 ^b^
**NTH-I**	8.16 ± 0.42 ^i^	1.88 ± 0.22 ^h^	7.34 ± 0.58 ^f^
**NTH-II**	3.09 ± 0.23 ^d^	0.85 ± 0.06 ^e,i^	2.83 ± 0.18 ^c^
**Fr.B1**	3.74 ± 0.24 ^e^	1.09 ± 0.12 ^j^	3.36 ± 0.32 ^d^
**Fr.B2**	0.98 ± 0.07 ^g^	0.48 ± 0.05 ^c,f^	1.56 ± 0.13 ^e^
**Fr.B3**	3.06 ± 0.15 ^d^	0.94 ± 0.06 ^i,j^	2.35 ± 0.22 ^c^

All values were the mean ± standard deviation (SD). EC50 (mg/mL) was defined as the concentration at which a sample caused a 50% decrease in the initial concentrations of DPPH•, HO•, and ${\text{O}}_{\text{2}}^{-}$•. (a–j) Values with different letters indicated significant differences in the same sample at different times (*p* < 0.05).

#### 2.2.2. Gel Filtration Chromatography of ATH-II and NTH-II

**Figure 2:**
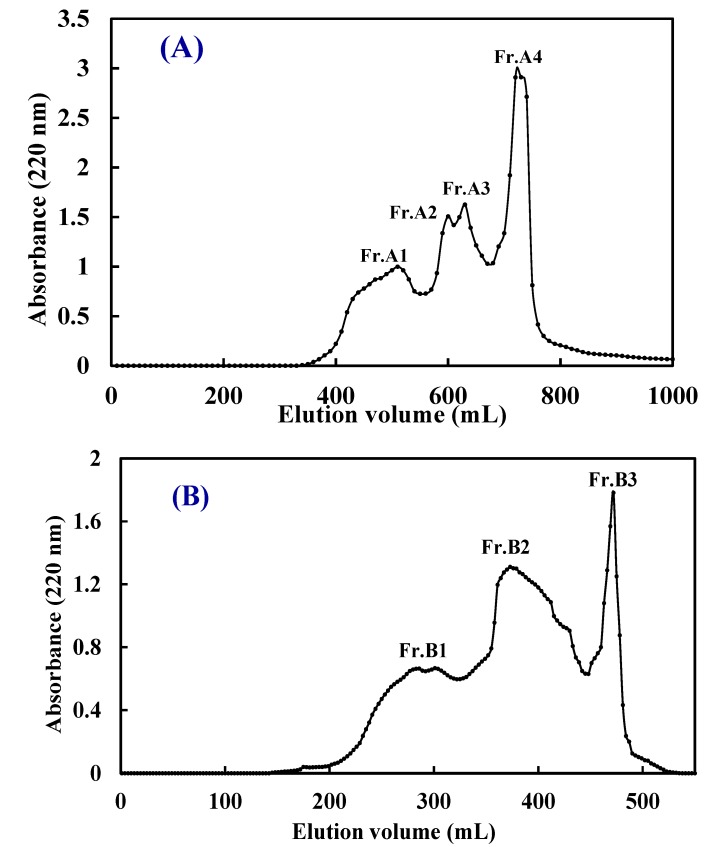
Elution profiles of ATH-II (**A**) and NTH-II (**B**) obtained by size exclusion chromatography on a Sephadex G-25 column.

Gel filtration is a method that enables the separation of substances with different molecular dimensions, and it has been widely used to separate peptides from protein hydrolysates [36]. In this study, ATH-II and NTH-II were further purified on a Sephadex G-25 column (Figure 2). As shown in Figure 2A, ATH-II was subsequently separated into four subfractions (Fr.A1 to Fr.A4). Fr.A3 showed significantly higher radical scavenging activities than the other ATH-II subfractions (*p <* 0.05) did, with EC50 values of 1.08 ± 0.08 (DPPH•), 0.22 ± 0.05 (HO•), and 1.31 ± 0.11 mg/mL (${\text{O}}_{\text{2}}^{-}$•) (Table 2). NTH-II was divided into three subfractions (Fr.B1 to Fr.B3). Fr.B2 showed significantly stronger DPPH•, HO•, and ${\text{O}}_{\text{2}}^{-}$• scavenging activities than the other two NTH-II subtractions (*P* <0.05) did, with EC50 values of 0.98 ± 0.07, 0.48 ± 0.05, and 1.56 ± 1.03 mg/mL, respectively (Figure 2B and Table 2). Moreover, the DPPH•, HO•, and ${\text{O}}_{\text{2}}^{-}$• scavenging activities of Fr.A3 were higher than those of Fr.B2.

### 2.3. Amino Acid and Peptide Compositions of Fr.A3 and Fr.B2

#### 2.3.1. Amino Acid Compositions of Fr.A3 and Fr.B2

The amino acid composition of any food protein hydrolysate plays a significant role in various physiological activities, which has also been observed for the antioxidant activities of protein hydrolysates [37]. The amino acid profiles of defatted STDM, ATH, NTH, Fr.A3 and Fr.B2 are shown in Table 3. The amino acid compositions, hydrophobic amino acid contents, and aromatic amino acid contents of ATH and NTH are similar to those of defatted STDM, which was rich in Glu, Asp, Leu, and Ala but lacked Cys, Pro, Trp, and Tyr. These results indicated that the hydrolysis process did not significantly alter the profile of the amino acids [32]. However, the amino acid compositions of Fr.A3 and Fr.B2 were different from those of the defatted STDM and its hydrolysates. Additionally, the hydrophobic and aromatic amino acid contents of these fractions were significantly higher than those of ATH and NTH. Pownall *et al.* confirmed that the higher contents of hydrophobic and aromatic amino acids facilitated the radical scavenging and metal chelating activities of protein hydrolysate fractions from pea seeds (*Pisum sativum* L.) [38]. Himaya *et al.* reported that hydrophobic amino acids facilitated interactions with hydrophobic targets, such as the cell membrane, and thereby, enhanced the bioavailability [2]. Additionally, hydrophobic peptides can protect against macromolecular oxidation by donating photons to reactive radicals. Mendis *et al.* reported that the high potency of HGPLGPL as an antioxidant could be derived from hydrophobic amino acids, which may facilitate greater interaction between the peptide and fatty acids [13]. Furthermore, aromatic amino acids increased the antioxidant activities of peptides and protein hydrolysates because they easily donate protons to electron-deficient radicals and maintain their stabilities *via* resonance structures and enhance radical scavenging activities [8,38,39,40]. Therefore, the high hydrophobic and aromatic amino acid contents of Fr.A3 and Fr.B2 might be an important reason for their high antioxidant activities.

**Table 3:** Amino acid compositions of Defatted STDM, ATH, NTH, Fr.A3 and Fr.B2 (expressed as residues/1000 residues).

Amino Acid	Defatted STDM	ATH	NTH	Fr.A3	Fr.B2
Asp (D)	98.2	96.9	97.3	79.9	91.2
Glu (E)	127.6	125.2	125.7	94.0	106.9
Ser (S)	71.4	70.5	70.8	65.2	60.8
Gly (G)	79.6	76.8	77.3	63.8	69.6
His (H)	75.4	74.7	74.6	81.1	73.9
Arg (R)	69.7	70.2	68.9	53.6	61.9
Thr (T)	48.5	47.7	48.3	36.5	41.2
Cys (C)	3.9	4.1	4.5	6.4	5.5
Tyr (Y)	11.3	11.7	12.6	24.0	22.5
Lys (K)	29.6	30.2	29.5	25.6	29.1
Ala (A)	79.4	80.1	80.5	89.8	86.1
Pro (P)	9.7	9.4	9.2	14.9	10.6
Val (V)	72.4	73.6	73.1	85.4	81.7
Met (M)	24.7	25.6	25.3	21.2	20.0
Ile (I)	53.9	54.7	53.4	78.7	70.1
Leu (L)	94.3	96.3	97.1	103.9	92.5
Trp (W)	10.8	12.4	11.6	26.4	21.9
Phe (F)	39.6	39.9	40.3	49.6	54.5
Total	1000	1000	1000	1000.0	1000.0
Aromatic amino acids	137.1	138.7	139.1	181.1	172.8
Hydrophobic amino acids	384.8	392	390.5	469.9	437.4

#### 2.3.2. Peptide Profiles and Compositions of Fr.A3 and Fr.B2

**Figure 3:**
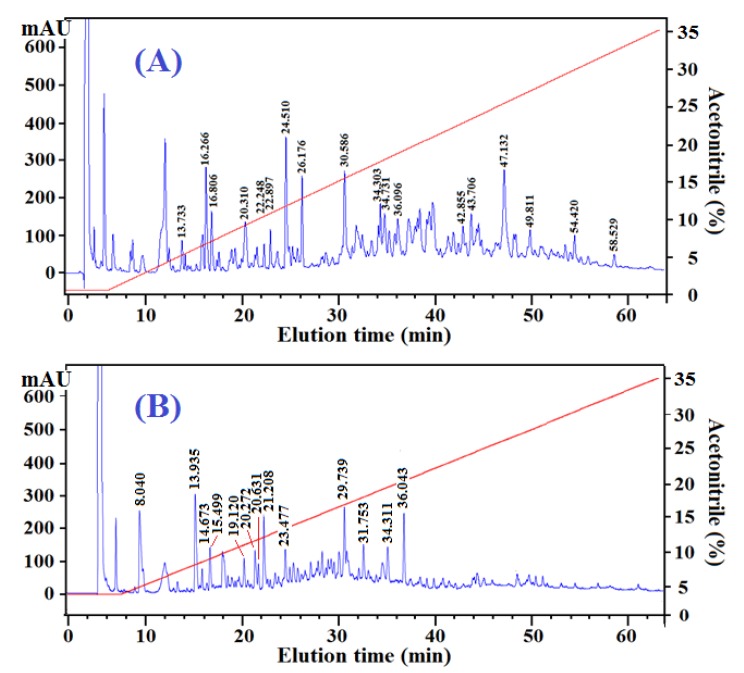
Elution profiles of Fr.A3 (**A**) and Fr.B2 (**B**) obtained by RP-HPLC on a Zorbax SB-C18 column (4.6 mm × 250 mm, Agilent, USA). Absorbance was measured at 220 nm.

In addition to the amino acid composition, peptide size and sequence are two other key factors that affect the activity of protein hydrolysates and peptides [8]. As shown in Table 1, the amino acid compositions of ATH and NTH were similar, but the radical scavenging activity of ATH was higher than that of NTH. Therefore, to further explain the difference in the activities of Fr.A3 and Fr.B2, their peptide profiles were obtained using reversed-phase high performance liquid chromatography (RP-HPLC) (Figure 3). Partial peptides in the two fractions were prepared, and their amino acid sequences were determined using a Procise Protein/Peptide Sequencer and Electrospray ionization mass spectrometry (ESI-MS) (Table 4). The activities of the peptides were evaluated based on their antioxidant scores, which are based on the presence and position of certain amino acid residues in the peptide sequences [32].

As shown in Figure 3, there are two distinct differences in the HPLC chromatograms of Fr.A3 and Fr.B2. Fr.A3 was composed of more peptides, and the components were primarily eluted between 10 and 60 min, whereas Fr.B2 had fewer peptides and was primarily eluted between 10 and 40 min. RP-HPLC utilizes a non-polar stationary phase and a moderately polar, aqueous mobile phase. With the stationary phase, the retention time is longer for molecules that are more non-polar, whereas polar molecules elute more readily. Therefore, based on their shorter retention time, the hydrophobic properties of the peptides in the Fr.B2 might be weaker than those of Fr.A3. This finding agreed with previous results indicating that the hydrophobic amino acid content of Fr.A3 was higher than that of Fr.B2 (Table 3).

Using RP-HPLC, 18 peptides from Fr.A3 and 13 peptides from Fr.B2 were prepared, and their amino acid sequences were determined (Table 4). The 18 peptides from Fr.A3 included five tripeptides, six tetrapeptides, six pentapeptides, and one hexapeptide, and the mean molecular weight was 524.78 kDa based on their theoretical MWs. The 13 peptides from Fr.B2 included three tetrapeptides, nine pentapeptides and one decapeptide, and the mean molecular weight was 585.91 kDa. These results indicated that the peptides from Fr.A3 had smaller molecular sizes and lower MWs.

**Table 4:** Potential antioxidant peptides and their antioxidant scores from Fr.A3 and Fr.B2, as identified by Procise Protein/Peptide Sequencer and ESI-MS.

No.	Retention Time (min)	Observed Mass (Da)	Calculated Mass (Da)	Amino Acid Sequence	Antioxidant Score
**Peptides from Fr.A3**					
A01	13.733	335.33	335.36	YGP	5.5
A02	16.266	482.42	482.44	YEGD	4.5
A03	16.806	463.53	463.55	QWM	7.5
A04	20.310	538.50	538.51	EYNN	4
A05	22.248	423.41	423.42	QNY	4.5
A06	22.897	331.30	331.33	QAGG	2
A07	24.510	429.45	429.47	QPW	8.5
A08	26.176	407.41	407.42	QFN	3
A09	30.586	660.69	660.72	DVIEW	9.5
A10	34.303	902.91	902.9	QYDEYW	9.5
A11	34.731	574.64	574.67	WVGTI	9.5
A12	36.096	563.57	563.60	DLYPG	5
A13	42.855	571.61	571.62	YVAGY	6
A14	43.706	489.49	489.52	DVWA	8
A15	47.132	528.58	528.60	QPVW	9.5
A16	49.811	681.73	681.74	QELHR	3
A17	54.420	619.69	619.71	EYIPV	7.5
A18	58.529	441.45	441.48	QPPT	3
**Peptides from Fr.B2**					
B01	8.040	446.39	446.41	QGGEG	2
B02	13.935	538.48	538.51	YENGG	4.5
B03	14.673	506.44	506.46	QESGS	2
B04	15.499	510.46	510.50	QYSGG	4
B05	19.120	521.49	521.52	EGYPG	4
B06	20.272	407.40	407.42	QFGG	3
B07	20.631	494.48	494.50	QFGGS	3
B08	21.208	464.45	464.47	QFGGG	3
B09	23.477	681.73	681.74	WGYAW	10
B10	29.739	742.83	742.86	YIVYW	12
B11	31.753	1214.21	1214.24	WGDAGGYYYY	10
B12	34.311	544.62	544.64	QILTA	3
B13	36.043	543.54	543.57	QPWN	8

### 2.4. Protective Effects of Fr.A3 and Fr.B2 on Lipid Peroxidation in a Linoleic Acid System.

In the radical scavenging experiments, Fr.A3 and Fr.B2 showed strong DPPH•, HO•, and ${\text{O}}_{\text{2}}^{-}$• scavenging activities. However, antioxidants have different mechanisms of action (*i.e.*, radical chain inhibition, metal chelation, oxidative enzyme inhibition or antioxidant enzyme cofactors). Therefore, the radical scavenging assay does not reflect the multiple mechanisms through which the samples may act as antioxidants to retard and/or inhibit lipid oxidation in a food system. In this section, the abilities of Fr.A3 and Fr.B2 to suppress lipid peroxidation in a linoleic acid system were investigated.

**Figure 4:**
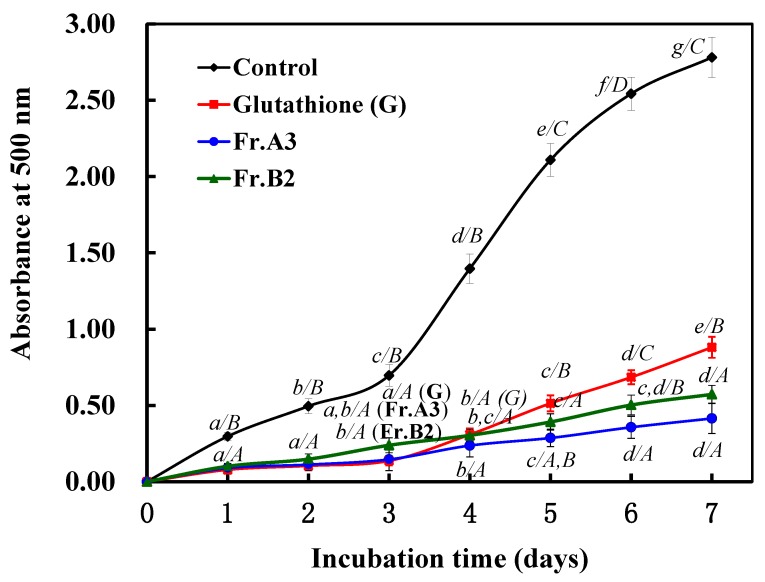
Lipid peroxidation inhibition assays of Fr.A3 and Fr.B2 in a linoleic acid oxidation system over seven days. The degree of linoleic acid oxidation was assessed by measuring the optical density at 500 nm at 24-h intervals. Glutathione was used as the positive control.

As shown in Figure 4, the control (without antioxidant) had the highest experimental absorbance value, indicating the highest degree of oxidation among the samples after a seven-day incubation period. Fr.A3, Fr.B2 and glutathione, however, effectively inhibited lipid peroxidation in the linoleic acid emulsion system for the entire seven days. From days 1–3, the inhibition activity of Fr.A3 was close to that of glutathione for lipid peroxidation in the test system, but the inhibition ability of Fr.A3 was stronger than that of glutathione from days 3–7. From days 1–4, Fr.B2 exhibited weaker inhibition activity than glutathione, but the inhibition activity of Fr.B2 was higher than that of glutathione from days 4–7. Over the entire testing period, Fr.B2 was less effective at inhibiting linoleic acid oxidation compared with Fr.A3.

## 3. Discussion

Bioactive peptides with 2–20 amino acids that are derived from food sources have received increased attention due to their more certain and efficient effects in the prevention or control of human diseases. Antioxidant peptides have received increased attention due to their significant role in the prevention and treatment of different disorders, such as atherosclerosis, cancer, diabetes mellitus, and coronary heart diseases [41,42]. Fish proteins have long been recognized for their nutritional and functional properties, the latter of which can be modified by physical, chemical or enzymatic treatments. Recently, research has focused on the liberation of bioactive peptides derived from fish proteins using enzyme technology, which involves milder processing conditions, relatively simpler reaction controls, and minimal byproduct formation [12,13]. Furthermore, the resulting peptides are intended for use as functional food ingredients to help maintain human health. However, the protein source, DH, amino acid composition, MWs, peptides sequences, and peptide compositions of the protein hydrolysates determine their bioactivities and functional properties [43]. At present, many studies have been performed on the antioxidant abilities of hydrolysates or bioactive peptides from seafood sources, including croceine croaker (*Pseudosciaena crocea*) muscles [4], sea urchin gonads [44], tilapia (*Oreochromis niloticus*) gelatin [6], monkfish (*Lophius litulon*) [13], Amur sturgeon skin [45], thornback ray [46], and bluefin leatherjacket (*Navodon septentrionalis*) heads [47].

In this experiment, five proteases were used to hydrolyze defatted STDM. ATH and NTH showed higher antioxidant activities than the other hydrolysates did. The results indicated that the antioxidant activities of the STDM protein hydrolysates were affected by the type of the enzyme used, which agrees with the report that the type of protease applied in hydrolysis is crucial to hydrolysate activity because of the different peptide bond cleavage patterns involved [18]. Moreover, the radical scavenging activities of the hydrolysates were positively correlated with the DH (%). The data suggested that the peptides in the different hydrolysates, which might have different chain lengths and amino acid sequences, contributed to radical scavenging to varying degrees. Therefore, ATH and NTH were most likely composed of more active peptides than the other hydrolysates did. These active peptides are electron donors that react with free radicals to convert them to more stable products and terminate the radical chain reaction. However, defatted STDM, ATH and NTH showed similar amino acid profiles, but their radical scavenging activities were different. These results indicated that the hydrolysis process did not significantly alter the profile of the amino acids [32,48] and that the differences in the activities may be caused by other factors, such as molecular size and amino acid sequences. Due to their free radical scavenging activities, ATH and NTH were further used for the preparation of the antioxidant peptides fractions.

Protein hydrolysates are composed of free amino acids and short-chain peptides that exhibit numerous advantages as nutraceuticals, functional foods or medicines. In fact, peptides and protein hydrolysates display different antioxidant activities depending on the peptide size, the amino acid sequence, and the presence of amino acids involved in oxidative reactions [13,49,50]. Therefore, high antioxidant peptides fractions (Fr.A3 and Fr.B2) were prepared using ultrafiltration and conventional chromatographic methods. Their amino acid compositions and peptide profiles were analyzed to determine the differences in their antioxidant activities. The results showed that Fr.A3 and Fr.B2 exhibited higher antioxidant effects than their original hydrolysates. Table 3 indicated that the amino acid compositions of Fr.A3 and Fr.B2 were different from those of the defatted STDM and its hydrolysates. The hydrophobic and aromatic amino acid contents of these fractions were significantly higher than those of ATH and NTH. Pownall *et al.* confirmed that the higher contents of hydrophobic and aromatic amino acids facilitated the radical scavenging and metal chelating activities of protein hydrolysate fractions from pea seeds (*P. sativum* L.) [38]. Himaya *et al.* reported that hydrophobic amino acids facilitated interactions with hydrophobic targets, such as the cell membrane, and thereby enhanced the bioavailability [2]. Additionally, hydrophobic peptides can protect against macromolecular oxidation by donating photons to reactive radicals. Mendis *et al.* reported that the high potency of HGPLGPL as an antioxidant could be derived from the hydrophobic amino acids, which may facilitate greater interactions between the peptide and fatty acids [13]. Furthermore, aromatic amino acids increased the antioxidant activities of peptides and protein hydrolysates because they easily donated protons to electron-deficient radicals, maintained their stabilities *via* resonance structures, and enhanced their radical scavenging activities [8,38,39,40]. Therefore, the high hydrophobic and aromatic amino acid contents of Fr.A3 and Fr.B2 might be important for their high antioxidant activities. Lipid peroxidation is a complex process involving the formation and propagation of lipid radicals and hydroperoxides in the presence of oxygen [51]. The strong lipid peroxidation inhibition activity of Fr.A3 in a linoleic acid system suggested that Fr.A3 could react with radicals in the system, including peroxyl radicals, and thereby inhibit the propagation cycle of lipid peroxidation. The amino acid profile of Fr.A3 (Table 3) indicated that Fr.A3 was rich in hydrophobic amino acids, which might lead to greater interactions between the peptides and the fatty acids. Moreover, histidine-containing peptides are active against lipid peroxidation because of an imidazole moiety, which may be involved in hydrogen donation for lipid radical trapping [40,52]. Therefore, we speculated that the hydrophobic amino acids and the His residues might simultaneously contribute to the higher antioxidant activity of Fr.A3.

Molecular weight is typically understood to be a key factor affecting the activities of peptides. Table 4 showed that the mean molecular weights of Fr.A3 and Fr.B2 were 524.78 kDa and 585.91 kDa based on their theoretical MWs, respectively. These results indicated that the peptides from Fr.A3 had smaller molecular sizes and lower MWs. These findings are consistent with a report stating that smaller antioxidants have a greater probability of engaging in more effective interactions with free radicals and inhibiting the propagation of lipid peroxidation. Additionally, they have a greater chance of crossing the intestinal barrier to exert biologically relevant functions. Using the method developed by Conway *et al.* [53], the antioxidant scores of the 31 peptides were calculated and shown in Table 4. Of the 18 peptide sequences from Fr.A3, 14 peptides contained one or more W and/or Y residues, which accounts for their high antioxidant scores. Furthermore, 10 peptides possessed a terminal W and/or Y residue, four peptides had a terminal P, M, I or V residue, and 12 peptides contained one or more H, K, P, F, V and/or I residues, which are also considered beneficial to the antioxidant activities of the peptides. Due to these particular amino acid residues in the sequences, the mean antioxidant score of the 18 peptides was 6.11. For the 13 peptide sequences from Fr.B2, seven contained one or more W and/or Y residues, four possessed a terminal W and/or Y residue, and seven peptides had one or more H, K, P, F, V and/or I residues, but no peptide had an N-terminal V or L residue or a C-terminal M residue. The mean antioxidant score of the 13 peptides from Fr.B2 was 5.27, which was lower than that of the 18 peptides from Fr.A3. This result was consistent with the data in Table 2, which indicated that Fr.A3 exhibited stronger radical (DPPH•, HO•, and ${\text{O}}_{\text{2}}^{-}$•) scavenging activities than Fr.B2 did. Moreover, these mean antioxidant scores also substantiated the data in Table 3 and the previous discussion, indicating that both amino acid composition and position in peptide sequences are important factors for the antioxidant activities of peptides.

## 4. Experimental Section

### 4.1. Materials

Skipjack tuna dark muscle (*K. pelamis*) (STDM) was provided by Zhejiang Hailisheng Group Co., Ltd. (Zhejiang, China). Trypsin, pepsin, papain, glutathione, trifluoroacetic acid (TFA), 1,1-diphenyl-2-picrylhydrazyl (DPPH), Sephadex G-25, and trinitrobenzene sulfonic acid (TNBS) were purchased from Sigma-Aldrich Trading Co., Ltd. (Shanghai, China). Alcalase and Neutrase were purchased from Novozymes Biotechnology Co., Ltd. (Tianjin, China). Acetonitrile (ACN) was purchased from Thermo Fisher Scientific Co., Ltd. (Shanghai, China). All other chemicals and reagents were of analytical grade and were obtained from Sinopharm Chemical Reagent Co., Ltd. (Shanghai, China).

### 4.2. Preparation of Protein Hydrolysates of STDM

The STDM was rinsed using tap water, pounded into a homogenate, and defatted as previously described [7]. The homogenate and isopropanol were mixed in a ratio of 1:4 (w/v) and stirred continuously for 4 h at 35 °C. Isopropanol was replaced every 2 h. The precipitate was collected by centrifugation at 9000 rpm for 15 min at 4 °C, freeze-dried and stored at −20 °C.

The defatted STDM was dissolved (10% w/v) in different buffer solutions and hydrolyzed for 4 h using each of the five proteases (listed below) at their optimal temperatures and pH conditions with an enzyme/substrate (E/S) ratio of 2% (w/w). The optimal conditions were as follows: trypsin (0.05 M Tris-HCl, pH 8.0, 37 °C), pepsin (0.05 M Gly-HCl, pH 2.0, 37 °C), papain (0.05 M phosphate, pH 6.5, 50 °C), Alcalase (0.05 M Gly-NaOH, pH 9.5, 50 °C) and Neutrase (0.05 M phosphate, pH 7.0, 60 °C). Enzymatic hydrolysis was terminated by heating for 10 min in boiling water, and the hydrolysates were centrifuged at 10,000 rpm for 15 min. The resulting supernatants were freeze-dried and stored at −20 °C until further analysis. The protein hydrolysates prepared using Alcalase and Neutrase were called ATH and NTH, respectively.

### 4.3. Determination of the Degree of Hydrolysis (DH)

The DH, defined as the percentage ratio of the number of peptide bonds broken to the total number of bonds per unit weight, was calculated according to a previously described method [54]. Hydrolysate (50 μL) was mixed with 0.5 mL of phosphate buffer (0.2 M, pH 8.2) and 0.5 mL of TNBS reagent (0.05%) freshly prepared by diluting with deionized water. After being incubated at 50 °C for 1 h in a water bath, the reaction in mixture was stopped by adding 1 mL of HCl (0.1 M) and incubated at 25 °C for 30 min. The absorbance was measured at 420 nm. l-Leucine was used as a standard. To determine the total amino acid content, defatted skate muscle was completely hydrolyzed with 6 M HCl with a sample/acid ratio of 1:100 at 120 °C for 24 h. DH (%) was calculated using the following equation:

DH = [(A_t_ − A_0_)/(A_max_ − A_0_)] × 100%

where, A_t_ is the number of α-amino acids released at time t, A_0_ is the number of α-amino acids in the supernatant at 0 h, and A_max_ is the total number of α-amino acids obtained after acid hydrolysis at 120 °C for 24 h.

### 4.4. Determination of Amino Acid Composition

The amino acid composition of samples was determined according to the method of Siswoyo *et al.* with slight modifications [52]. To determine the amino acid composition, freeze-dried collagen was dissolved in distilled water to obtain a concentration of 1 mg/mL, and an aliquot of 50 mL was dried and hydrolyzed in vacuum-sealed glass tube at 110 °C for 24 h in the presence of 6 M HCl, which contained 0.1% phenol. Norleucine (Sigma Aldrich, Inc., St. Louis, MO, USA) was used as an internal standard. After hydrolysis, the samples were again vacuum-dried, dissolved in application buffer and injected into an automated amino acid analyzer (HITACHI 835-50 Amino Acid Analyzer, Japan). The determinations were performed in triplicate, and the data corresponded to the mean values. The standard deviations were lower than 2% in all cases.

### 4.5. Antioxidant Activities

#### 4.5.1. Radical (DPPH•, HO•, and ${\text{O}}_{\text{2}}^{-}$•) Scavenging Assays

The radical (DPPH•, HO•, and ${\text{O}}_{\text{2}}^{-}$•) scavenging assays were performed as described by Wang *et al.* with ascorbic acid (AA) as positive control [3]. The results were expressed as the median effective concentration (EC50), which was defined as the concentration at which a sample caused a 50% decrease in the initial concentrations of DPPH•, HO•, and ${\text{O}}_{\text{2}}^{-}$•.

##### HO• Scavenging Activity

HO• scavenging activity was measured using the method developed by Wang *et al.* [3]. In this system, HO• is generated by the Fenton reaction. HO• can oxidize Fe^2+^ to Fe^3+^, and only Fe^2+^ can combine with 1,10-phenanthroline to form a red compound (1,10-phenanthroline-Fe^2+^). It shows maximum absorbance at 536 nm. The concentration of HO• is reflected by the degree of decolorization of the reaction solution. Briefly, 1,10-phenanthroline solution (1.0 mL, 1.865 mM) and the sample (2.0 mL) were added into a screw-capped tube and mixed. The FeSO_4_·7H_2_O solution (1.0 mL, 1.865 mM) was then pipetted into the mixture. The reaction was initiated by adding 1.0 mL H_2_O_2_ (0.03% v/v). After incubation at 37 °C for 60 min in a water bath, the absorbance of the reaction mixture was measured at 536 nm against a reagent blank. Reaction mixture without any antioxidant served as the negative control, and mixture without H_2_O_2_ served as the blank. HO• scavenging activity (HRSA) was calculated by the following formula:

HRSA(%) = [(A_s_ − A_c_)/(A_b_ − A_c_)] × 100%

where A_s_ is the sample absorbance; A_c_ is the control group absorbance; and A_b_ is the blank absorbance.

##### DPPH• Scavenging Activity

DPPH• scavenging activity was tested as previously described [3]. Two milliliters of deionized water containing different concentrations of sample (from 0.125–2.0 mg/mL) were placed in the cuvettes, and then 500 μL of ethanol solution of DPPH (0.02%) and 1.0 mL of ethanol were added. The control containing DPPH solution without sample was also prepared. In blank, DPPH solution was replaced with ethanol. The antioxidant activity of the sample was evaluated using the relative amount of inhibition of DPPH• with the following equation:

DPPH• scavenging activity (%) = (A_c_+A_b_-As)/A_c_ × 100%

where A_s_ is the sample absorbance; A_c_ is the control group absorbance; and A_b_ is the blank absorbance.

##### ${\text{O}}_{\text{2}}^{-}$•Scavenging Activity

The ${\text{O}}_{\text{2}}^{-}$• scavenging activity of the sample was investigated by the previous method [3]. In the experiment, ${\text{O}}_{\text{2}}^{-}$• was generated in 1 mL of nitrotetrazolium blue chloride (2.52 mM), 1 mL of NADH (624 mM), and 1 mL of different concentrations of samples (from 0.065–1.0 mg/mL). The reaction was initiated by adding 1 mL of phenazine methosulfate solution (120 μg/mL) to the reaction mixture. The absorbance was measured at 560 nm against the corresponding blank after 5 min incubation at 25 °C. The capacity of scavenging ${\text{O}}_{\text{2}}^{-}$• was calculated using the following equation: 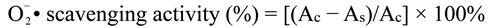 where A_s_ is the absorbance with sample; and A_c_ is the absorbance without sample.

#### 4.5.2. Lipid Peroxidation Inhibition Assay

The protective effect of the isolated peptides on lipid peroxidation in a linoleic acid model system was measured based on a previously described method [55]. Briefly, a sample (0.5 mg/mL) dissolved in 10 mL of phosphate buffer (0.05 M, pH 7.0) was added to 10 mL of 99.5% ethanol containing 0.13 mL of linoleic acid, and the final volume was adjusted to 25 mL with distilled water. The mixture, stored in a sealed screw-cap conical tube, was incubated at 40 °C in the dark. The degree of linoleic acid oxidation was measured at 24-h intervals for seven days using the ferric thiocyanate method, in which 0.1 mL of the reaction mixture was mixed with 4.7 mL of ethanol (75%), 0.1 mL of ammonium thiocyanate (30%), and 0.1 mL of ferrous chloride (0.02 M) in 3.5% HCl. After 3 min, the degree of color development, which was indicative of linoleic acid oxidation, was measured at 500 nm.

#### 4.5.3. Antioxidant Score

For subsequent identification of potentially antioxidant buttermilk protein derived peptides, antioxidant score was established for scoring peptide sequences based on the presence and position of certain amino acid residues. The antioxidant score was calculated according to the method described by Conway *et al.*: Trp (W) within the amino acid sequence, 5 points; Tyr (Y) within the amino acid sequence, 2 points; short sequence length (2–10 residues), 2 points; His (H), Lys (K), Pro (P), Phe (F), Val (V), or Ile (I) within the sequence, 1 point; Tyr (Y), Trp (W), Val (V), or Leu (L) residues at N-terminus, 0.5 point; and Trp (W), Tyr (Y), and Met (M) residues at C-terminus, 0.5 point [53].

### 4.6. Separation of Antioxidant Peptides

#### 4.6.1. Ultrafiltration

Ultrafiltration is a technique for separating molecules in solution based on molecular size. It is widely used to concentrate target fractions from protein hydrolysates on a MWCO membrane. ATH and NTH were fractionated at 4 °C by ultrafiltration (8400, Millipore, Hangzhou, China) with 3 kDa molecular weight cutoff (MWCO) membranes (Millipore, Hangzhou, China), appropriate for the laboratory scale. The peptide fractions (ATH-I and ATH-II, NTH-I and NTH-II) were collected and lyophilized for separation in the following step.

#### 4.6.2. Gel Filtration Chromatography

Gel filtration, an effective method for separating bioactive substances with different molecular dimensions, was used for the group separation of the protein hydrolysates. In this study, the ATH-II and NTH-II solutions (5 mL, 20.0 mg/mL) were separately loaded onto a Sephadex G-25 column (2.6 × 80 cm) pre-equilibrated with deionized water and eluted at a flow rate of 1 mL/min. Each eluted fraction (3 mL) was collected and monitored at 220 nm. Four subfractions (Fr.A1, Fr.A2, Fr.A3 and Fr.A4) from ATH-II and three fractions (Fr.B1, Fr.B2, and Fr.B3) from NTH-II were collected and lyophilized for further studies.

#### 4.6.3. Reversed-Phase High Performance Liquid Chromatography (RP-HPLC)

The fractions (Fr.A3 and Fr.B2) with high radical scavenging activities obtained from the Sephadex G-25 column were further separated by RP-HPLC (Agilent 1260 HPLC, Agilent Ltd., Santa Clara, CA, USA). The Fr.A3 and Fr.B2 solutions (20 μL, 100.0 μg/mL) were injected separately into a Zorbax SB-C18 column (4.6 mm × 250 mm, Agilent, USA) using a linear gradient of ACN (0%–0% for 6 min and 0%–100% over 100 min) at a flow rate of 1.0 mL/min. The elution peaks were monitored at 220 nm, and the fractions were collected manually, lyophilized and stored until further analysis.

### 4.7. Identification of the Peptide Sequences and Molecular Masses

The antioxidant peptides of SP-A and SP-B were subjected to N-terminal amino acid sequencing on an Applied Biosystems 494 protein sequencer (Perkin Elmer Wallace Inc., Akron, OH, USA) according to previous methods [3]. Edman degradation was performed according to the standard program supplied by Applied Biosystems.

Accurate molecular masses of SP-A and SP-B were determined using a Q-TOF mass spectrometer (Micromass, Waters, Los Angeles, California, USA) coupled with an electrospray ionization (ESI) source based on a previous method developed by Luo *et al.* [16]. Ionization was performed in positive mode with a capillary voltage of 3500 V. Nitrogen was maintained at 40 psi for nebulization and 9 L/min at 350 °C for evaporation temperature. The data were collected in centroid mode from *m*/*z* 100–2000.

### 4.8. Statistical Analysis

All experiments were conducted in triplicate (*n* = 3), and the values were expressed as mean ± standard deviation (SD). An ANOVA test using the SPSS 19.0 software was performed to analyze the experimental data. Duncan’s multiple range test (*p* < 0.05) was used to measure the significant differences between the means of the parameters.

## 5. Conclusions

In this study, dark muscle from skipjack tuna (*K. pelamis*) was hydrolyzed using five separate proteases, including pepsin, trypsin, Neutrase, papain and Alcalase. Two hydrolysates, ATH and NTH, prepared using Alcalase and Neutrase, respectively, showed the strongest radical scavenging activities. Using ultrafiltration and gel filtration chromatography, two fractions, Fr.A3 and Fr.B2, were prepared from ATH and NTH, respectively, and their antioxidant activities, amino acid compositions and peptide profiles were investigated. The results indicated that Fr.A3 and Fr.B2 could effectively inhibit lipid peroxidation in the linoleic acid model system and scavenge DPPH•, HO•, and ${\text{O}}_{\text{2}}^{-}$•. Smaller molecular sizes, hydrophobic and aromatic amino acid residues, and amino acid sequences were the key factors determining the antioxidant activities of the proteins, hydrolysates and peptides. Moreover, the present study provided useful information for increasing the commercial value of STDM byproducts as multifunctional ingredients, and suggested new ways to increase its recognition as a natural source of antioxidants that are capable of preventing oxidation in food systems.
